# Spine day 2012: spinal pain in Swiss school children– epidemiology and risk factors

**DOI:** 10.1186/1471-2431-13-159

**Published:** 2013-10-05

**Authors:** Brigitte Wirth, Christina Knecht, Kim Humphreys

**Affiliations:** 1 Institute of Human Movement Sciences and Sport, ETH Zurich, Wolfgang Pauli Str. 27, 8093 Zurich, Switzerland; 2 Chiropractic Department, University of Zurich and Uniklinik Balgrist, Forchstr. 340, 8008 Zurich, Switzerland

**Keywords:** Adolescence, Risk factor, Spinal pain

## Abstract

**Background:**

The key to a better understanding of the immense problem of spinal pain seems to be to investigate its development in adolescents. Based on the data of Spine Day 2012 (an annual action day where Swiss school children were examined by chiropractors on a voluntary basis for back problems), the aim of the present study was to gain systematic epidemiologic data on adolescent spinal pain in Switzerland and to explore risk factors per gender and per spinal area.

**Method:**

Data (questionnaires and physical examinations) of 836 school children were descriptively analyzed for prevalence, recurrence and severity of spinal pain. Of those, 434 data sets were included in risk factor analysis. Using logistic regression analysis, psycho-social parameters (presence of parental back pain, parental smoking, media consumption, type of school bag) and physical parameters (trunk symmetry, posture, mobility, coordination, BMI) were analyzed per gender and per spinal area.

**Results:**

Prevalence of spinal pain was higher for female gender in all areas apart from the neck. With age, a steep increase in prevalence was observed for low back pain (LBP) and for multiple pain sites. The increasing impact of spinal pain on quality of life with age was reflected in an increase in recurrence, but not in severity of spinal pain. Besides age and gender, parental back pain (Odds ratio (OR)=3.26, p=0.011) and trunk asymmetry (OR=3.36, p=0.027) emerged as risk factors for spinal pain in girls. Parental smoking seemed to increase the risk for both genders (boys: OR=2.39, p=0.020; girls: OR=2.19, p=0.051). Risk factor analysis per spinal area resulted in trunk asymmetry as risk factor for LBP (OR=3.15, p=0.015), while parental smoking increased the risk for thoracic spinal pain (TSP) (OR=2.83, p=0.036) and neck pain (OR=2.23, p=0.038). The risk for TSP was further enhanced by a higher BMI (OR=1.15, p=0.027).

**Conclusion:**

This study supports the view of adolescent spinal pain as a bio-psycho-social problem that should be investigated per spinal area, age and gender. The role of trunk asymmetry and passive smoking as risk factors as well as the association between BMI and TSP should be further investigated, preferably in prospective studies.

## Background

Spinal pain is an immense socio-economic problem in most of the industrialized countries in terms of pain, disability and time off of work. The key to understanding the development of spinal pain in adults seems to be in understanding its development in adolescents. It has been shown that the prevalence of low back pain (LBP) doubles from the age of 12 to 15 [1,2] and was reported to be 30% for females and 26% for males at the age of 14 years [3]. Lifetime prevalence approximates adult levels around the age of 18 [4] resulting in the fact that at the age of 20, half of the adolescents have experienced LBP [1,4]. Also neck pain is common in teenagers. Its prevalence is similar to LBP (24% for males and 34% for females at the age of 14 years) [3]. These findings are extremely important because adolescent spinal pain has been shown to be highly associated with spinal pain in adulthood. LBP in childhood, for example, results in a fourfold risk of LBP in adulthood [5]. Although some studies emphasize that spinal pain in adolescents is benign with minimal impact [6] and warn against medicalization of the problem [7], the majority of studies state that adolescent spinal pain is an important public health issue and that the focus of research, prevention and treatment in this area should be changed from the adult to the young population [5].

Research into adolescent spinal pain can roughly be divided into three main sub-areas, namely epidemiological studies focusing on prevalence, studies on (psycho-social and physical) risk factors and longitudinal studies that focus on the course or on prevention/treatment of spinal pain throughout adolescence. As a major limitation, many studies in the past did not differentiate the location of ‘back pain’. Thus, a detailed assessment of spinal pain in terms of location was recommended by recent studies [4,8,9]. Furthermore, in order to make the various studies on spinal pain comparable, standardization of definition of adolescent back pain by assessing recurrence and severity was recommended [10].

Regardless of area, spinal pain seems to be more common in girls than in boys [4,9,11] and a daily computer use of more than two hours [12], but not obesity [13], seems to increase the risk for spinal pain. For lumbar pain specifically, LBP of one or both parents [14,15], anxiety and depression [14,16], TV consumption and smoking, but not body weight or fitness level have been reported as risk factors [7,17-19]. Controversial results were found with regard to physical parameters [7,20,21] and it was thus suggested that psychosocial factors were more important than mechanical factors for LBP in young populations [22]. However, the investigation of physical risk factors was mainly focused on physical activity, body mass index (BMI), school bag weight, muscle strength and (lumbar) spine mobility [7,20]. Information on risk factors for thoracic spinal pain (TSP) and neck pain (NP) is scarce. While back pack weight and chair height at school have been identified as risk factors for TSP [9], genetic influences [23] and psychosomatic symptoms [11] were associated with NP.

Since 2006, on the occasion of the WHO’s International Spine Day, the Swiss Chiropractic Association (ChiroSuisse) have organized an annual action day (Spine Day) where Swiss school children were examined as a service on a voluntary and free of charge basis for back problems. Based on the data of Spine Day 2012, the aim of the present study was twofold; first to gain systematic, although not representative epidemiologic data on adolescent spinal pain in Switzerland by optimizing the data collection according to the literature guidelines as described above. The second aim was to explore risk factors for spinal pain per gender and per spinal area (LBP, TSP, NP, pain in more than one spinal area) using the common risk factors based on the literature and by complementing the physical investigation with measures for trunk symmetry, posture and coordination.

## Methods

### Participants

Ethical approval for this study was given by the Ethics committees of all cantons of Switzerland that required an Ethics proposal for this type of study (BS/BL, LU, SG, VD) and by the Ethics committee of ETH Zurich. Participants were recruited throughout Switzerland by advertisements in print and electronic media and by flyers that were distributed in chiropractic practices. Seventy-seven chiropractic practices volunteered for Spine Day 2012. Altogether, 1040 children and adolescents participated. In compliance with the decision of the responsible Ethics committees, the parents (or the legal representatives), but not the children had to give their signature on the questionnaire if they agreed that the (anonymized) data of the children were included in this study. Since the present study focused on school children aged 6 to 16 years, the exclusion criteria for this study were being younger than 6 or older than 16 years and missing a parental or guardian signature. Thus, data of 104 children younger than 6 years and of 18 adolescents older than 16 years were not analyzed. Information about age was missing on 11 questionnaires. These children were excluded from further analyses.

Consequently, 836 questionnaires (382 boys, 449 girls, 5 with missing gender information; mean age = 10.3±2.8 years, mean height = 1.45±0.17 m, mean weight = 38.3±13.5 kg) were included in the data analysis.

### Procedure

Prior to Spine Day 2012, the participating chiropractors were informed of the study during a meeting organized by ChiroSuisse association. On Spine Day 2012 (November 10), the participants and their parents or representatives filled in the questionnaire. Apart from demographic information (sex, age), the questionnaire covered the following topics: 1) lifetime prevalence of spinal pain [8] (LBP, NP, TSP, pain in more than one spinal area), 2) recurrence of spinal pain in the last month [4] (once, sometimes, often, daily), 3) severity of spinal pain (visual analog scale (VAS) from 0 to 10), 4) consequences of spinal pain (reduction of leisure activities, school absence, seeing a doctor or chiropractor, taking medication), 5) frequency and duration of TV/computer activities, 6) type of school bag (backpack, shoulder bag, briefcase), 7) smoking habits of participant and his parents, 8) spinal pain history of parents. Question 4 on consequences of spinal pain was ambiguous and could not be analyzed. As a very few participants smoked themselves, only the influence of parental smoking habits was analyzed.

The physical investigation by the chiropractors started with measuring the body height and weight. It further consisted of an assessment of posture (Matthiass arm-raising test [24]), which tested a child’s ability to hold his arms for 60 seconds in 90 degrees flexion without changing posture towards thoracic kyphosis and/or lumbar lordosis. Coordination was tested by asking the children to stand 10 seconds on each leg, once with eyes open and once with eyes closed [25]. Since several studies have reported reduced balance performance in adults with LBP [26], particularly with closed eyes [27], the single leg stance with closed eyes was of interest and was included in a further analysis. Furthermore, trunk and rib cage symmetry was assessed by the Adam’s forward bend test: The chiropractor noted whether a participant demonstrated a rib hump while bending forwards from a standing position. This test is the most widely used test in school scoliosis screening [28]. Trunk asymmetry and the diagnosis of scoliosis have so far been reported to increase the risk for LBP by one study each [29,30], but the significance of these variables need further investigation [30,31]. Lastly, mobility was tested by measuring finger to floor distance when bending forward while in a standing position [32]. The participants were asked to bend forward as far as possible with knee, arms and fingers extended and the chiropractor measured the distance between the fingertips and the floor (finger-floor distance, FFD). This test is considered to give information about trunk mobility by assessing combined spine and pelvic mobility [32]. In the present study, no differentiation was made between a participant who touched the floor with the finger tips or with flat hands. Furthermore, the chiropractors investigated spinal mobility (range of motion), static deformities in the lower extremity (hips, knees and feet) and tested whether palpation of the vertebrae was painful. These data, however, were not included in the analyses.

### Data analysis and statistics

For analysis of spinal pain epidemiology, the children were divided into three age categories representing three different school grades in Switzerland (there were slight variations between cantons): 6–9 years (N=346, mean age = 7.6±1.1 years, mean height = 1.30±0.09 m, mean weight=27.2±6.0 kg), 10–12 years (N=278, mean age = 11.0±0.8 years, mean height = 1.50±0.09 m, mean weight=40.3±9.1 kg), 13–16 years (N=212, mean age = 14.0±1.0 years, mean height = 1.66±0.09 m, mean weight=54.0±10.0 kg). Data were analyzed per gender using descriptive statistics. For the determination of risk factors, logistic regression analyses (forced entry/enter method) were conducted including the following categorical (binary) factors (coding 0/1): Parental spinal pain (no/yes; no differentiation whether mother and/or father has pain), smoking parents (no/yes; no differentiation whether mother and/or father smokes), type of school bag (backpack/briefcase or shoulder bag), daily TV or computer activity (<1 hour/>1 hour; frequency and duration were multiplied), Adams forward bending test (absence/presence of rib hump), Matthiass test (no posture change after 60 seconds/posture change from beginning or within 60 seconds), single leg stance for 10 seconds with closed eyes (possible on both legs/not possible on one or both legs), gender (male/female). Age, BMI and FFD were analyzed as continuous variables. In order to minimize the influence of age, FFD was normalized and expressed as a percentage of height. Risk factors profiles were determined 1) for spinal pain in general (per gender) and 2) for pain per spinal area (LBP, TSP, NP, pain in more than one spinal area). For the analysis per spinal area, the participants with pain in the area of interest were compared to the children without pain in any spinal area. For epidemiologic analyses, data sets with missing values were excluded from the corresponding analyses only (available case-analysis). For the risk factor analyses, only complete data sets could be included (complete case analysis) (N=434, 211 boys, mean age=10.4±2.8 years, mean height = 1.46±0.17 m, mean weight=39.0±13.9 kg). All analyses were conducted using SPSS 20. The significance level was set at p<0.05.

## Results

### Epidemiology

#### Prevalence, recurrence and severity of spinal pain

At the age of 6 to 9 years, the prevalence of spinal pain was approximately one third, comparable in both boys and girls. At the age of 10 to 12 years, the prevalence was almost double in girls, while a similar increase was observed in boys after the age of 12 years (Table 1). This increase in spinal pain prevalence could mainly be attributed to LBP and to spinal pain in more than one area, while the prevalence of NP and TSP remained relatively stable.

**Table 1:** Lifetime prevalence of spinal pain

	**6-9 (N=332)**		**10-12(N=271)**		**13-16(N=205)**		**6-16(N=808)**	
	**Male N=156 (%)**	**Female N=176 (%)**	**Male N=120 (%)**	**Female N=151 (%)**	**Male N=97 (%)**	**Female N=108 (%)**	**Male N=373 (%)**	**Female N=435 (%)**
No spinal pain	63.5	69.9	62.5	39.7	40.2	22.2	57.1	47.6
Spinal pain	36.5	30.1	37.5	60.3	59.8	77.8	42.9	52.4
Neck pain	14.7	9.7	6.7	14.6	13.4	7.4	11.8	10.8
Low back pain	7.1	5.7	9.2	16.6	18.6	24.1	10.7	14.0
Thoracic pain	5.8	4.5	2.5	9.3	7.2	9.3	5.1	7.4
Multiple pain localization	9.0	10.2	19.2	19.9	20.6	37.0	15.3	20.2

The recurrence of spinal pain within the last month before the Spine Day assessment steadily increased with age (Table 2). The percentage of unique complaints decreased, while the prevalence of children with regular pain increased. At the age of 13 to 16 years, 44% of the children complained about spinal pain once a week and 9% complained about daily pain.

**Table 2:** Recurrence of spinal pain in the last month

	**6-9 years N=99(%)**	**10-12 years N=126(%)**	**13-16 years N=134(%)**	**6-16 years N=359(%)**
Once	42.4	29.4	28.4	32.6
Once a week	38.4	46.0	44.0	43.2
Repeatedly a week	15.2	17.5	18.7	17.3
Daily	4.0	7.1	9.0	7.0

The severity of spinal pain was moderate in all age groups and only slightly increased with age. The children between 6 and 9 years indicated on average a score of 3.7 points (SD=1.9; range 0.5-9) on the VAS scale. At the age of 10 to 12 years, mean VAS score was 3.9 points (SD=1.9; range 0.5-10), which increased to 4.5 points at the age of 13 to 16 years (SD=1.8; range 1–10).

### Risk factors

#### Spinal pain in general

A summary of the results of all tested possible risk factors for adolescent spinal pain is shown in Table 3. The regression analyses showed that female gender is, with an odds ratio (OR) of 1.9 (p=0.003), a risk factor for spinal pain in children (Table 4). An increase in age was associated with spinal pain regardless of gender, with OR of 1.3 (p=0.001) for boys and 1.4 (p<0.001) for girls. Similarly, the exposure to parental smoking demonstrated a consistent association with an increased risk for spinal pain, with OR of 2.4 for boys (p=0.020) and 2.2 for girls (p=0.051). In contrast, BMI, mobility (FFD), posture (Matthiass Test) and coordination (single leg stance) did not show any consistent association with spinal pain (OR values around 1.0). No distinct pattern emerged for the factors parental spinal pain, trunk symmetry and type of schoolbag. Spinal pain in mother and/or father, which was present in the vast majority of parents (Table 3), tripled the risk for spinal pain in girls (OR=3.3, p=0.027), but did not seem to be of relevance in boys (OR =1.5, not significant). Similarly, the presence of a positive Adam’s sign, indicating trunk asymmetry, significantly increased the risk for spinal pain only in girls (OR=3.4, p=0.027), but not in boys (OR=1.0, not significant). Conversely, the use of a briefcase or shoulder bag significantly decreased the risk for spinal pain in girls (OR=0.027, p=0.005), but showed only a trend for an increased risk in boys (OR=3.6, p=0.092).

**Table 3:** Possible risk factors for spinal pain (boys N=211, 90/121 with/without spinal pain; girls N=223, 121/102 with/without spinal pain)

**Risk factor**	**Risk factor categories**	**Gender**	
		**m**	**f**
Parental pain	Yes (Child P/child no P)	192 (83/109)	190 (109/81)
	No (Child P/child no P)	19 (7/12)	33 (12/21)
Smoking parents	Yes (Child P/child no P)	41 (24/17)	43 (27/16)
	No (Child P/child no P)	170 (66/104)	180 (94/86)
Schoolbag	Backpack (Child P/child no P)	200 (82/118)	183 (82/101)
	Briefcase/shoulder bag (Child P/child no P)	11 (8/3)	40 (20/20)
Computer per week	<1h/day (Child P/child no P)	165 (69/96)	183 (94/89)
	>1h/day (Child P/child no P)	46 (21/25)	40 (27/13)
Adam’s Sign	Positive/Asymmetry (Child P/child no P)	23 (10/13)	26 (19/7)
	Negative/Symmetry (Child P/child no P)	188 (80/108)	197 (95/102)
Matthiass Test	Posture deficit (Child P/child no P)	74 (28/46)	74 (37/37)
	No posture deficit (Child P/child no P)	137 (62/75)	149 (84/65)
Single leg stance with eyes closed	Reduced (Child P/child no P)	57 (22/35)	53 (25/28)
	Ok on both legs (Child P/child no P)	154 (68/86)	170 (96/74)
Normalized finger floor distance	Mean child with spinal pain (SD)	6.27 (5.46)	3.79 (4.88)
	Mean child without spinal pain (SD)	5.59 (5.49)	3.36 (4.61)
BMI	Mean child with spinal pain (SD)	18.03 (2.95)	18.34 (3.55)
	Mean child without spinal pain (SD)	17.22 (2.66)	17.24 (3.09)

BMI=body mass index; f=female; m=male; P=pain; SD=standard deviation.

**Table 4:** Risk factors for spinal pain

**Spinal pain**		**All children (N=434, N with pain=211) R**^**2 **^**=0.20**			**Boys (N=211, N with pain=90) R**^**2 **^**=0.17**			**Girls (N=223, N with pain=121) R**^**2 **^**=0.26**		
**Parameter**	**df**	**Exp (B)**	**95% CI Exp (B)**	**p**	**Exp (B)**	**95% CI Exp (B)**	**p**	**Exp (B)**	**95% CI Exp (B)**	**p**
Gender		**1.93**	**1.25-2.97**	**0.003**						
Age	1	**1.31**	**1.19-1.44**	**<0.001**	**1.27**	**1.11-1.46**	**0.001**	**1.40**	**1.20-1.62**	**<0.001**
Spinal pain parents	1	**2.36**	**1.20-4.68**	**0.013**	1.53	0.50-4.68	0.454	**3.26**	**1.31-8.09**	**0.011**
Smoking parents	1	**2.11**	**1.25-3.57**	**0.005**	**2.39**	**1.14-5.00**	**0.020**	2.19	1.00-4.80	0.051
Computer/TV per week	1	0.97	0.57-1.65	0.909	0.74	0.36-1.55	0.426	1.24	0.54-2.88	0.616
Schoolbag	1	0.68	0.33-1.37	0.275	3.61	0.81-16.00	0.092	**0.27**	**0.11-0.68**	**0.005**
Trunk asymmetry	1	1.68	0.85-3.30	0.135	1.03	0.39-2.74	0.958	**3.36**	**1.15-9.84**	**0.027**
Posture	1	1.01	0.64-1.59	0.971	0.95	0.50-1.83	0.887	1.15	0.59-2.25	0.687
Coordination	1	0.99	0.60-1.64	0.978	0.99	0.49-1.99	0.971	1.04	0.50-2.19	0.917
Mobility	1	1.00	0.96-1.04	0.927	1.01	0.96-1.07	0.711	0.99	0.93-1.05	0.705
BMI	1	1.01	0.94-1.09	0.738	0.99	0.87-1.12	0.817	1.05	0.95-1.17	0.358

#### Pain per spinal area

Increasing age, female gender and the presence of a rib hump (Adam’s sign) emerged as risk factors for LBP. A positive Adam’s sign tripled the risk (p=0.015), while female gender increased the risk by a factor 2.5 (p=0.014) and every year of age by a factor of 1.4 (p<0.001) (Table 5). Parental spine pain showed a tendency to influence LBP in children (OR=3.0), although this was not significant (p=0.068). As for TSP, female gender (OR=4.3, p=0.003), smoking parents (OR=2.8, p=0.036) and a higher BMI (OR=1.2, p=0.027) significantly increased the risk for pain in this area (Table 6). Neck pain significantly increased with age (OR=1.2, p=0.050) and seemed to be present more often in children whose parents smoked (OR=2.2, p=0.038), while the presence of parental spinal pain (OR=3.3, p=0.073) showed a tendency to increase the risk for adolescent neck pain (Table 7). The presence of spinal pain in more than one area could only be related to increasing age (OR=1.4, p<0.001) and female gender, which doubled the risk (OR=2.0, p=0.036) (Table 8).

**Table 5:** Risk factors for low back pain

**Low back pain**		**All children with low back pain and those without spinal pain in any area (N=279, N with pain=56) R**^**2 **^**=0.26**		
**Parameter**	**df**	**Exp (B)**	**95% CI Exp (B)**	**p**
Age	1	**1.38**	**1.18-1.62**	**<0.001**
Gender	1	**2.45**	**1.20-5.00**	**0.014**
Spinal pain parents	1	2.98	0.92-9.61	0.068
Smoking parents	1	1.55	0.66-3.62	0.314
Computer/TV per week	1	1.39	0.62-3.10	0.425
Schoolbag	1	0.44	0.15-1.36	0.154
Trunk asymmetry	1	**3.15**	**1.25-7.92**	**0.015**
Posture	1	1.19	0.54-2.62	0.673
Coordination	1	0.48	0.17-1.34	0.161
Mobility	1	0.94	0.88-1.01	0.110
BMI	1	0.98	0.86-1.13	0.822

**Table 6:** Risk factors for thoracic back pain

**Thoracic back pain**		**All children with thoracic back pain and those without spinal pain in any area (N=253, N with pain=30) R**^**2 **^**=0.23**		
**Parameter**	**df**	**Exp (B)**	**95% CI Exp (B)**	**p**
Age	1	1.17	0.96-1.43	0.114
Gender	1	**4.31**	**1.62-11.44**	**0.003**
Spinal pain parents	1	2.46	0.66-9.16	0.179
Smoking parents	1	**2.83**	**1.07-7.48**	**0.036**
Computer/TV per week	1	1.09	0.38-3.11	0.872
Schoolbag	1	0.41	0.10-1.63	0.203
Trunk asymmetry	1	0.27	0.02-3.12	0.292
Posture	1	0.38	0.12-1.24	0.110
Coordination	1	1.46	0.48-4.39	0.504
Mobility	1	0.99	0.91-1.09	0.879
BMI	1	**1.15**	**1.02-1.31**	**0.027**

**Table 7:** Risk factors for neck pain

**Neck pain**		**All children with neck pain and those without spinal pain in any area (N=273, N with pain=50) R**^**2 **^**=0.10**		
**Parameter**	**df**	**Exp (B)**	**95% CI Exp (B)**	**p**
Age	1	**1.17**	**1.00-1.36**	**0.050**
Gender	1	1.46	0.74-2.88	0.274
Spinal pain parents	1	3.25	0.90-11.82	0.073
Smoking parents	1	**2.23**	**1.05-4.74**	**0.038**
Computer/TV per week	1	0.49	0.18-1.32	0.156
Schoolbag	1	1.16	0.40-3.30	0.788
Trunk asymmetry	1	0.73	0.19-2.75	0.642
Posture	1	0.67	0.32-1.44	0.306
Coordination	1	1.17	0.54-2.55	0.692
Mobility	1	1.02	0.96-1.08	0.481
BMI	1	0.98	0.86-1.11	0.721

**Table 8:** Risk factors for spinal pain in more than one spinal area

**Back pain in more than one spinal area**		**All children with spinal pain in more than one area and those without spinal pain in any area (N=297, N with pain=74) R**^**2 **^**=0.23**		
**Parameter**	**df**	**Exp (B)**	**95% CI Exp (B)**	**p**
Age	1	**1.44**	**1.25-1.67**	**<0.001**
Gender	1	**1.98**	**1.05-3.74**	**0.036**
Spinal pain parents	1	2.16	0.81-5.79	0.125
Smoking parents	1	1.75	0.85-3.63	0.131
Computer/TV per week	1	1.08	0.53-2.19	0.840
Schoolbag	1	0.47	0.17-1.32	0.151
Trunk asymmetry	1	1.85	0.76-4.46	0.173
Posture	1	1.67	0.90-3.12	0.105
Coordination	1	1.20	0.61-2.38	0.600
Mobility	1	1.03	0.97-1.08	0.385
BMI	1	1.01	0.90-1.13	0.904

## Discussion

The first aim of this study was to present epidemiologic data on adolescent spinal pain based on Spine Day 2012. Although this study was not representative, several findings from the literature could be confirmed by these data. While lifetime prevalence of neck and thoracic spine pain were relatively low and showed less age dependency, a steep increase in LBP with age was observed. This increment seemed to start earlier in girls than in boys which is in line with previous literature [1]. While prevalence of LBP was comparable in boys and girls at the age of 6 to 9 years, it steeply increased in the age group of 10 to 12 years in girls, but not before the age of 13 to 16 in boys. In the age group of 13 to 16 years, prevalence was higher in girls. An increase in prevalence with age and higher prevalence in girls has been reported before [33]. Similar results were found for pain in multiple areas of the spine. Prevalence markedly increased in both genders between 6 to 9 and 10 to 12 years and further increased in girls. The high prevalence of spinal pain in multiple areas might be of clinical relevance, since an earlier review suggested that children with multiple pain localization should be carefully observed [33]. Altogether, these findings strongly support the postulation that prevalence data of spinal pain should be reported per spinal area, age and gender [4]. The increasing impact of spinal pain on quality of life with age was subjectively reflected in recurrence, but not in severity of spinal pain. In the age group of 13 to 16 years, 9% of the adolescents complained about daily pain and every fifth adolescent reported spinal pain several times a week. This might be of importance since it has been shown that persistent LBP at an early age in particular is a strong predictor of persistent LBP later in life [5]. Severity of pain, in contrast, only slightly increased with age. In all age groups, it was moderate according to pain definitions in children and adolescents [34], which corresponds to findings from other studies [35].

Spinal pain in general and in all areas apart from the neck seems to be a major problem particularly for the female gender. In line with former studies, which reported female gender to be a significant risk factor for LBP (OR values between 1.11 and 2.43 [19,29,36]), girls emerged from this study as having more than double the risk for LBP (OR = 2.45) compared to boys. TSP in particular seemed to be associated with female gender, which confirms the results of a recent review on TSP [9]. In contrast, female gender was not a risk factor for neck pain in the present study. Earlier studies found an association between female gender and neck pain, but these studies included older participants [3,11]. An interesting twin study on genetic influences on neck pain in early adolescence, however, did not show sex-specific genetic effects [23]. In this study, the presence of spinal pain in parents emerged as risk factor for spinal pain particularly in girls and for LBP and neck pain, although in tendency only. An association between LBP and presence of LBP in at least one parent has been reported in several studies [20,33]. The fact that this study found the association to be of importance for girls in particular raises the question on the possible role of genetic or psychosocial factors. It might be hypothesized that girls are influenced by the parental role model more than boys. A surprising finding of the present study was that parental smoking seemed to increase the risk for spinal pain for both genders similarly as well as for TSP and neck pain. Many other studies have reported active smoking to be a risk factor for back pain [7,33], presumably rather by reflecting psychosocial problems than by being a causal factor [7,37]. As for second-hand smoke, a study that found a positive relationship between active smoking and back pain in adolescents raised the hypothesis that second-hand smoke might promote back pain in adolescents similarly than active smoking, probably on the basis of biologic mechanisms affecting disc health [38]. Indeed, promotion of disc degeneration by exposure to passive smoking has been shown in rats [39]. The only study, however, which was found that investigated the role of passive smoking on LBP in childhood did not find an association [15]. Nevertheless, the role of passive smoking in the development of spinal pain needs further investigation. The role of media consumption (TV, computer) is controversial in the current research literature. Nevertheless, it is generally thought that two hours of daily media consumption seems to be the critical threshold for the development of back pain [33] and musculoskeletal symptoms in general [12]. In this study, one hour of daily media consumption was chosen as the threshold which might explain the fact that media consumption did not emerge as risk factor. Consequently, in a future study, two hours of media consumption should be used as the cut-off point. According to the present study, the type of schoolbag was reported to have no influence on LBP [7,15,29]. However, the presence of back pain in general was conversely influenced for girls and boys in the present study. Since the vast majority of children, particularly boys, used backpacks, this result has to be interpreted very cautiously. Nonetheless, the investigation of risk factors per gender might reveal results that might remain hidden when observing both genders together.

Gender specific risk factor analysis might reveal important differences for physical parameters. Trunk asymmetry emerged as a risk factor for spinal pain in girls and for LBP. Trunk asymmetry and being diagnosed with scoliosis was reported to increase the risk for LBP [29,30]. Although trunk asymmetry as a possible risk factor for the development of back pain has to be validated first [31], this finding should be of interest for further, preferably prospective studies. In contrast, no relation to spinal pain was found for the Matthiass Test which is supposed to assess posture deficiencies and mobility measured by FFD. This corresponds to earlier findings [40]. Nevertheless, it must be borne in mind that measuring mobility by FFD is a rather unspecific test that assesses combined trunk mobility (pelvis and spine) [32]. A more specific assessment of spine mobility might reveal different results. The Matthiass Test, however, which is conversely debated due to lack of reliability [41], should be eliminated from further screening protocols. The same is applicable for the single leg stance test. Center of pressure excursion was shown to be enhanced in adults with LBP [26]. However, it should be investigated first whether this applies also for children and, if so, whether this test is sensitive enough to detect such changes. Lastly, in accordance with the literature [20,33], BMI did not show an association with LBP. Surprisingly, however, BMI was identified as a risk factor for TSP in the present study, which is contrary to the results of a recent study on obesity as risk factor for adolescent musculoskeletal pain [13]. Although it cannot be ruled out that this result was found by chance and thus needs confirmation, this finding shows again the necessity to investigate spinal pain per area as well as to conduct further research on risk factors for TSP, as current data are scarce [9].

### Limitations

The participation on Spine Day was optional which almost certainly led to a selection bias. Thus the prevalence rates in this study may not represent the overall prevalence of adolescent back pain in the general Swiss population which is a major limitation of this project. A representative study that assesses the burden of adolescent spinal pain in the Swiss population is desirable. A further major limitation of the present study was the large amount of missing data which resulted in the loss of some important information. Only about half of the participants could be included in the risk factor analysis which made it impossible to investigate risk factors per spinal area and gender. Although the analysis for spinal pain in general per gender and the analysis per spinal area for both genders revealed some interesting findings, further studies should investigate in more detail the areas of spinal pain and gender. Another limitation was the fact that the questionnaire which was used was not validated. However, to our knowledge, no validated questionnaire focusing on children and adolescents was available at that time. Meanwhile, the “Young Spine Questionnaire” has recently been published [42], although it has not yet been translated and validated in German. Nevertheless, in future, the question on consequences of spinal pain should be reworded, since this is an important measure for severity [10]. Similarly, psychological factors such as anxiety and depression were not assessed, although they have been reported to be significant risk factors [14,33].

## Conclusions

In summary, the present study supports the view of adolescent spinal pain as a bio-psycho-social problem that needs further research in particular of comparing the spinal area of pain with age and gender. The role of passive smoking and trunk asymmetry as risk factors for spinal pain as well as the association between BMI and pain in the thoracic spine also need further investigation, preferably in prospective studies.

## Appendix 1: Questionnaire

1. Do you have now or did you ever have pain in your back or neck?

2. If you ever have experienced pain: How often did you have pain in the last month?

3. If you ever have experienced pain: How severe was your pain (VAS)?

4. If you ever have experienced pain: What did you do against your pain?

5. How often per week do you sit in front of the computer or the TV?

6. For how long at a time do you sit in front of the computer or the TV?

7. What type of schoolbag do you use?

8. Do you smoke or do your parents smoke?

9. Do your parents suffer from back or neck pain?

## Appendix 2: Clinical examination

1. Height

2. Weight

3. Back profile inspection: static posture

4. Feet and knee inspection: static deviations

5. Matthiass arm-raising test: hold arms for 60 seconds in 90 degrees flexion

6. Single leg stance for 10 seconds with open and closed eyes

7. Adam’s forward bend test: rib hump

8. Finger floor distance in forward bending

9. Mobility (range of motion): neck, thoracic spine, lumbar spine

10. Palpation: painful spinosus processes

## Competing interests

The authors declare that they have no competing interests.

## Authors’ contributions

BW made substantial contribution to the conception of the questionnaire of Spine Day 2012, was involved in data analysis, wrote the manuscript and gave final approval. CK was involved in data analysis, revised the manuscript and gave final approval. KH was involved in conception of Spine Day 2012, revised the manuscript and gave final approval. All authors read and approved the final manuscript.

## Pre-publication history

The pre-publication history for this paper can be accessed here:

http://www.biomedcentral.com/1471-2431/13/159/prepub
